# The Use of Cannabinoids in the Treatment of Inflammatory Bowel Disease (IBD): A Review of the Literature

**DOI:** 10.7759/cureus.36148

**Published:** 2023-03-14

**Authors:** Basil N Nduma, Kelly A Mofor, Jason Tatang, Chukwuyem Ekhator, Solomon Ambe, Ekokobe Fonkem

**Affiliations:** 1 Internal Medicine, Merit Health Wesley, Hattiesburg, USA; 2 Gastroenterology, Texas Tech Paul L. Foster School of Medicine, El Paso, USA; 3 Gastroenterology, Sam Houston State University, Huntsville, USA; 4 Neuro-Oncology, New York Institute of Technology, College of Osteopathic Medicine, Old Westbury, USA; 5 Neurology, Baylor Scott & White Health, McKinney, USA; 6 Neuro-Oncology, Baylor Scott & White Health, Temple, USA

**Keywords:** inflammatory bowel disease, crohn’s disease (cd), ulcerative colıtıs, cannabinoid, cannabis sativa, cannabis

## Abstract

Around the world, about 15 to 40% of individuals with inflammatory bowel disease (IBD) rely on cannabis and cannabinoids to reduce the need for other medications, as well as increase appetite and reduce pain. Whereas more and more patients continue to report benefits accruing from cannabis and cannabinoid usage in IBD, agreement relative to the use of cannabis and its derivatives in IBD remains unclear. This paper reviewed the interplay between cannabinoid use and IBD disease treatment, remission, or symptom relief. The study was conducted from a systematic review perspective. It involved consulting literature from published original research articles, noting outcomes, and performing a meta-analysis to identify trends and draw conclusions. The selected articles were those that had been published in a 10-year period ranging between 2012 and 2022. The motivation was to ensure recency and also relevance to contemporary scientific research and clinical environment practices. Indeed, the Preferred Reporting Items for Systematic Reviews and Meta-Analyses (PRISMA) framework helped in answering the focal question of the investigation, which revolved around whether cannabinoids are beneficial to IBD treatment and to what extent. The aim of using this protocol was to ensure the satisfaction of the article exclusion and inclusion criteria, as well as ensure the utilization of articles directly contributing to the central subject under investigation. In the findings, it was established that on the one hand, cannabinoid usage in IBD treatment comes with promising results as reported in the majority of the selected studies which reported reduced clinical complications which were assessed using Mayo scores, Crohn’s Disease Activity Index (CDAI) score, weight gain, enhanced patient health perception, Lichtiger Index and Harvey-Bradshaw Index or general wellbeing. On the other hand, cannabinoid use remains questionable because evidence of high quality is yet to surface vividly, especially in terms of the mode of administration and the appropriate dose. It is also notable that the findings were characterized by a state of high heterogeneity in terms of the study designs of the studies that were selected, disease activity indices, the duration of treatment by different scholarly researchers, the difference in the modes of administration of cannabinoid and cannabis by different researchers, variations in cannabis dosage, differences in the selected studies’ inclusion criteria, and variations in their case definitions. The implication is that whereas the efficacy of cannabinoid use in IBD treatment was reported in most studies, outcome generalizability from the review was highly likely to be restricted. In the future, it is recommended that randomized controlled trials center, set universal parameters for IBD treatment using cannabis and cannabinoids to determine intervention safety and effectiveness as well as having homogenous outcomes that can be compared between different studies. In so doing, the appropriate dose and ideal mode of administration of cannabis and its derivatives might be discerned, ensuring relevance based on patient characteristics such as gender and age, as well as the appropriate administration mode and dose as per IBD symptom severity.

## Introduction and background

Inflammatory bowel disease (IBD) is a broad term used to describe a group of conditions that cause chronic inflammation of the digestive tract. IBD may affect as many as 200 persons in every 100,000 adults in the context of the United States (U.S) as well as 400 persons in every 100,000 adults in the context of the United Kingdom (UK) [1,2]. The two dominant and primary IBD types are ulcerative colitis (UC) and Crohn’s disease (CD). From the literature, the increasing incidence of IBD is attributed to the consumption of xenobiotics, reduced intake of vitamins and fiber, a diet rich in fats and sugars, stress, and genetic factors [3,4].

For these recurrent chronic conditions, however, effective treatment and management options are yet to be documented. In most cases, therapies that have been developed have come with several side effects, translating into an increase in healthcare costs on the part of patients. In the majority of patients, drugs such as corticosteroids tend to be effective, but the results of the therapies do not prove beneficial in other patients. Furthermore, the therapies have been associated with reactions due to medication infusion procedures, malignancy secondary to immunosuppression, bone marrow suppression, and opportunistic infections [5]. Hence, unconventional treatments can help to induce or maintain remission, also proving inexpensive. Mostly, physicians have explored the possible role of cannabinoids or their derivatives in a quest to discern their therapeutic adjuvant possibility [4,6].

Since ancient times, Cannabis sativa has been utilized for recreational or therapeutic purposes. It contains aromatic hydrocarbons in the form of terpenes and cannabinoids, whose location is mostly in the plant’s trachoma cavity. Associated with anti-inflammatory and antioxidant properties, the compounds have been observed to be beneficial, serving as therapeutic options for diseases such as cancer, schizophrenia, anxiety, and chronic pain. Whether patients with IBD can benefit and the extent to which patients with IBD can benefit from the plant and its associated derivatives becomes an area worth investigating [7-9]. Given that the misuse of Cannabis sativa can cause serious health issues, its use continues to draw much controversy. In some of the previous scholarly studies, however, IBD patients proving refractory to conventional therapies have been documented to benefit from treatment through Cannabis sativa and its derivatives [10]. The purpose of this review is to examine the relationship between the use of cannabinoids and the treatment of IBD. Specifically, it investigates the impact and suitability of using cannabis as a treatment option for IBD.

## Review

Materials and methods

**Figure 1:**
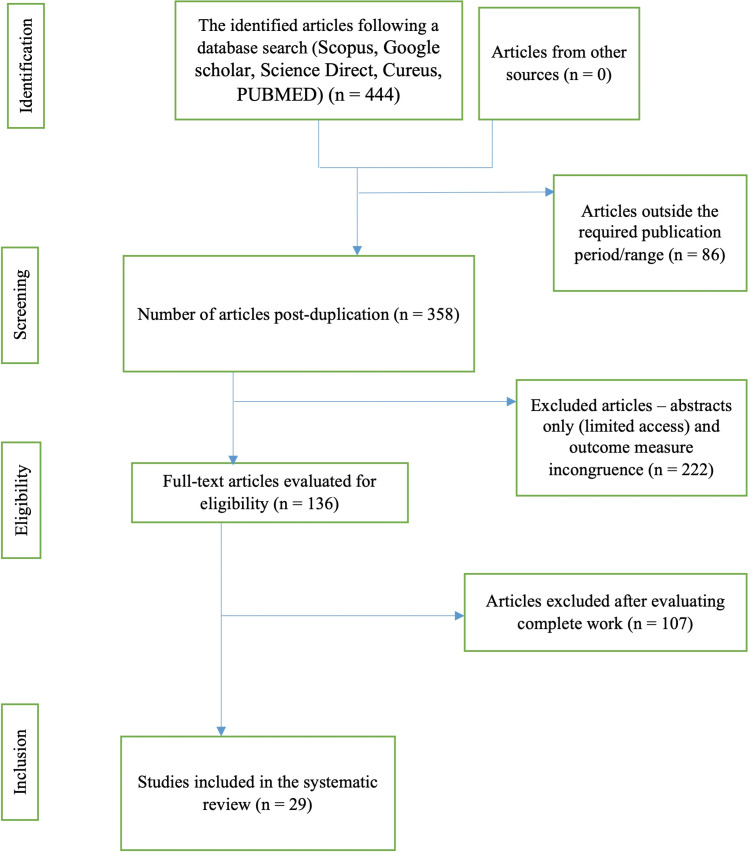
An illustration of the Preferred Reporting Items for Systematic Reviews and Meta-Analyses (PRISMA) protocol flowchart

This research adopted a systematic approach to reviewing the literature, which included examining original research articles and taking note of their outcomes to identify patterns and form conclusions. The study was conducted in accordance with the Preferred Reporting Items for Systematic Reviews and Meta-Analyses (PRISMA) recommendations to ensure that a rigorous methodology was followed. A key inclusion criterion was that selected studies had to have been published in the past decade and focused specifically on the effectiveness of cannabinoids in treating IBD. The aim of the criterion was to ensure recency in the published research, hence relevance to contemporary healthcare, expert opinions, and clinical practice. With an electronic search strategy on the focus, certain keywords were used to get ideal articles. They included IBD, cannabinoids, Cannabis sativa, tetrahydrocannabinol (THC), and cannabidiol. Eligible studies involved randomized controlled trials (RCT) including preclinical in vivo research studies touching on cannabinoid usage and the resulting treatment effectiveness or otherwise for IBD patients. The key databases from which the articles were drawn included Scopus, Google Scholar, Science Direct, and PubMed. Articles selected included those that were published between 2012 and 2022 in the English language, emphasizing original reviews and research papers. With eligible studies identified, the extraction of data involved a data extraction table, which then paved the way for describing study characteristics systematically, including their designs, results, and healthcare implications. To adhere to the principle of intellectual property rights, which governs scientific research, authors whose works were consulted were cited accordingly. The figure below highlights the procedure through which articles were included or excluded from the study based on the designed search strategy and article identification, screening, eligibility, and inclusion processes. Figure 1 below outlines the PRISMA flow chart of the current study.

Table 1 below offers a summary of the selected studies and their resulting insights, as well as implications in relation to the subject under investigation.

**Table 1:** Systematic Review Summary IBD: Inflammatory Bowel Disease; UC: Ulcerative Colitis; CD: Crohn's Disease; GPR: G-protein coupled receptor; CD: Cluster of Differentiation; THC: Tetrahydrocannabinol; ROS: Reactive Oxygen Species; CBG: Cannabigerol; FO: Fish Oil; CAuNP: Core Gold Nanoparticles; CAgNP: Core Silver Nanoparticles; NLC: Nanostructured Lipid Carrier

Author(s)	Year	Results	Research Implications
Pellino et al.	(2020) [1]	Cannabinoids were observed to be effective and safe in mitigating symptom severity in patients with IBD.	The study recommended the need for future studies to focus on varying levels of dosage and the impact on patient recovery from IBD disease.
Mak et al.	(2020) [2]	The addition of statin/aspirin did not yield additional benefits concerning chemoprevention.	In Chinese IBD patients, the clinical benefit of statin/aspirin addition during chemoprevention is yet to be established.
Li et al.	(2019) [3]	The Pathway Activation for the Single Sample technique was found to be reliable in predicting IBD precisely.	In the future, significant features in cannabinoids that could inform their efficacy for use with IBD patients are an area worth investigating.
Hansen et al.	(2019) [4]	The use of cannabis for self-medication in IBD persons was found to exhibit increased vulnerability to substance abuse.	Before using cannabis for IBD symptom treatment, screening for vulnerability to substance misuse and mental health comorbidities is key.
Papamichael et al.	(2020) [5]	Immunomodulator addition was found to increase drug concentrations and attenuate immunogenicity, hence anti-TNF agents’ pharmacokinetic profile improvement in IBD patients.	Combination therapy during cannabinoid use with IBD patients is an area worth considering in clinical environments.
Kafil et al.	(2019) [6]	In adults with UC and CD, no firm conclusions arose relative to cannabinoid and cannabis effectiveness and safety.	There is a need for further examination to ascertain the efficacy of the selected compounds.
Downer	(2020) [7]	Terpenes were found to exhibit anti-inflammatory properties.	Terpenes could be considered for use in ongoing cannabinoid-based therapies in IBD patients.
Weinberger et al.	(2020) [8]	Cannabis was found to exacerbate IBD, compounded by its increasing use in patients with depression.	Early screening for comorbidities is key when considering the use of cannabinoids and cannabis in IBD patients.
Andersen et al.	(2020) [9]	Cannabis use was avowed to predict levels of GPR15^+^CD3^+^CD4^+^ helper T cells	The efficacy of cannabinoids in terms of safety and effectiveness was documented, but larger samples ought to be considered in the future to ascertain the results.
Kienzl et al.	(2020) [10]	Cannabis was found to steer improvements in health-related quality of life for IBD patients, but the ability to reduce inflammatory markers remained a dilemma.	Given that heavy opioid use may come with higher mortality and opioid dependency, future clinical trials ought to focus on the magnitude of the dosage and ascertain levels that come with optimal patient outcomes.
Becker et al.	2020 [11]	The results of this investigation demonstrated the efficacy of delta-9-tetrahydrocannabinol in attenuating anti-CD40-induced colitis and colitis-associated colon cancer.	In the future, the study’s insights can form a foundation for the eventual examination of tissues from different sites to determine delta-9-tetrahydrocannabinol effects, including in the colon’s mucosal barrier sites.
Wang et al.	2022 [12]	In the findings, it was reported that zein-WP nanoparticles are superior in cannabinoid protection against UV.	The implication for future clinical practice is that the findings could inform the process of zein-WP nanoparticle encapsulation, hence improvements in cannabinoid release in vitro and also its antioxidant activity.
Borrelli et al.	(2013) [13]	Cannabigerol was found to attenuate murine colitis, as well as reduce the formation of reactive oxygen species in intestinal epithelial cells.	In IBD patients, Cannabigerol could be considered for clinical use or application.
Irving et al.	2018 [14]	The aim was to investigate the effect of botanical extracts rich in cannabinoids on IBD symptom severity. The results demonstrated that in individuals who tolerate the extracts, therapeutic benefits are felt. However, multiple limitations characterized the interpretation, including the failure to give insight into the implications for persons with drug intolerance.	The results could be used in the future to discern the impact of botanical extracts rich in cannabinoids on IBD symptoms in severe cases.
Naftali et al.	(2013) [15]	Significant clinical and steroid-free benefits were noted following the use of THC-rich cannabis for a short course of eight weeks.	In the future, studies with larger patient groups could be used to ascertain these findings.
Naftali et al.	2021 [16]	This study sought to investigate the impact of THC-rich cannabis usage on a short-term treatment basis. In UC patients, the results demonstrated beneficial clinical effects.	Future clinical practices could build on these results to seek to give insight into the degree of anti-inflammatory improvement and any moderating role brought about by potential laboratory markers.
Naftali et al.	2017 [17]	The safety of cannabinoids in moderately active Crohn’s disease patients was documented.	The study recommended the need for future studies to focus on a larger number of patients and also assess the moderating effect of the dosage level.
Nallathambi et al.	2017 [18]	When administered in low concentration, cannabinoid poses anti-inflammatory activity. However, the side effect is that it comes with dose-dependent cytotoxic activity, especially in high concentrations.	The results could be used to guide the treatment of colon tissues in IBD patients.
Cocetta et al.	2021 [19]	Cannabinoid as a compound was observed to yield promising results in relation to the treatment of intestinal inflammatory status via ROS production inhibition.	These findings are crucial for guiding the restoration of the permeability of the epithelium
Couch et al.	2019 [20]	With palmitoylethanolamide and cannabidiol implementation, human colon permeability is reduced.	The implication of the results can be felt in disorders involving gut permeability increase, with IBD unexceptional
Pagano et al.	2021 [21]	When CBG’s per se inactive dose, cannabinoid, and FO were combined, promising effects entailing intestinal anti-inflammation were reported.	The findings inform how, in the colon and in the serum, phytocannabinoid levels could be altered and yield positive patient outcomes.
Silvestri et al.	2020 [22]	With the co-administration of cannabinoids in low concentration and fish oil, the compounds were found to yield reductions in colon inflammation.	In the future, the findings can be used to determine the mechanism of action that explains Akkermansia muciniphila elevation.
Singh et al.	2012 [23]	Cannabis sativa extracts were investigated to determine how they could be used for economical and rapid nanoparticle green synthesis, with findings depicting the extracts’ effectiveness in curbing the formation of biofilms.	The results could be used in the future to guide how to prevent CAgNPs, C-AuNPs, and F-AuNPs, from forming.
Pagano et al.	2019 [24]	The authors strived to assess the effect of cannabinoid compounds on cytokine expression in pediatric groups with colonic biopsies due to UC. The results saw cannabinoids exert intestinal anti-inflammatory effects in the study subjects.	In future clinical environments, the results might be useful in prompting the examination of the extent to which cannabinoids might reduce cytokine expression in the rest of the population, eventually supporting clinical decision-making.
Hoffenberg et al.	2018 [25]	The motivation of the investigation involved understanding the effect of marijuana on IBD suppression in young adults and adolescents, with findings suggesting that whereas the product is perceived to be beneficial, testing and screening are key to mitigating adverse events.	Indeed, the results could help in the future to develop guidelines for informing the needed formulations for addressing IBD in young adults and adolescents.
Matarazzo et al.	2021 [26]	This study sought to uncover the role of in-situ gelling hydrogels in driving cannabinoids as drugs that are highly lipophilic. The results suggested that the hydrogels are not suitable, but promising formulations exist in the form of CBD-NLC dispersions, especially through CBD’s nasal administration.	The results could be used in the future to inform optimal formulations through which better cannabinoid compound-based interventions might help to address IBD in patients.
Harvey et al.	2014 [27]	The correlation between cannabinoid ligands and the mitigation of mucosal damage was the central purpose of the study, with the efficacy of cannabinoids documented.	The study’s inferences could aid in the future in determining cannabinoid compound formulations that could aid in restoring human colon functioning.
Tartakover Matalon et al.	2021 [28]	The impact of cannabis on the eCB tone was investigated. In UC patients, the beneficial effects of the compound were documented, including symptom relief.	The study was essential by paving the way for the future understanding of how cannabis may affect the quality of life in patients with different demographic features from both the clinical implications and anti-inflammatory impact perspectives.
Nso et al.	2021 [29]	In patients with IBD, cannabinoids were found to yield promising outcomes such as reduced clinical complications and better general well-being.	The study pointed to the need for future investigations to be conducted in terms of randomized controlled trials to gain further insight.

It has been observed and documented that the incidence and prevalence of IBD have been on the rise globally [11]. While genetic factors are known to contribute to the development of IBD, the available evidence suggests that their direct impact is less likely over time. As a result, there has been a growing focus on investigating the moderating role of external factors in the development of IBD. Previous scholarly investigations have explored a variety of external factors, including the immune system, the interaction between the organism and commensal microorganisms, and environmental factors [11]. One of the specific focal areas investigating the interplay between cannabinoids and IBD has been the assessment and provision of therapeutic proposals relative to its capacity to bring about symptom relief in IBD patients. The botanical species Cannabis sativa’s derived natural molecules, phytocannabinoids, have been investigated as alternatives for IBD treatment [12]. In the findings, it has been ascertained that in small cohort studies and preclinical models involving human subjects, phytocannabinoids play the role of endocannabinoid system modulation, hence great promise for IBD. However, it is worth noting that such evidence remains superficial especially from the methodological perspective, pointing to the criticality of greater robustness in the investigations. For example, the adequacy of the samples drawn from the sampling frame remains unexplained, putting outcome validity and reliability at stake.

In some retrospective studies, cannabis has been observed to relieve IBD symptoms, with prospective studies also suggesting notable improvements in patients with UC and CD after utilizing inhaled cannabis, including quality of life improvement [13]. One of the randomized studies centered on 75 persons diagnosed with UC, with 39 participants completing the study [14]. Targeting individuals with mild to moderate disease severity (Mayo score 4-10), the evaluation lasted 10 weeks, with 5-amino-salicylic acid on the focus. On the one hand, the limitation of the study is that the treatment period was short-term and low tolerance was evident. On the other hand, the chief strength of the study was that it increased the understanding and led to the insight that cannabidiol-rich botanical extract comes with beneficial effects relative to symptom relief in persons diagnosed with UC. When the participants’ health-related quality of life was also assessed, the results demonstrated that the extract poses a better response, suggesting that the botanical extract’s effect was promising. However, the extent to which the extract could be effective in patients with severe symptoms remained unaddressed. Similarly, the impact of any external factors in the environment that could play a moderating role or effect was not highlighted vividly, including the impact of lifestyle alteration alongside the pharmacotherapeutic intervention.

One of the recent prospective studies focused on 21 patients with Crohn’s Disease Activity Index (CDAI) > 200 whose mean age stood at 40±14 years. In the study, cannabis was given to these CD patients who did not respond to therapy with steroids, immune modulators, or anti-tumor necrosis factor α agents. These patients were given cannabis two times each day in the form of a placebo (cannabis flowers) or cigarette treatment (11.5 mg tetrahydrocannabinol) [15]. There were 10 patients in the placebo group and 11 patients in the cigarette treatment group. The treatment period stretched to eight weeks. Out of the 11 participants in the established Crohn’s disease treatment group, 10 patients demonstrated clinical improvement including three patients in this group who were completely weaned of steroid dependency and five patients in this group who achieved complete remission (CDAI score < 150). Hence, in the group using cannabis rich in tetrahydrocannabinol, close to 90% of the population exhibited a notable CDAI decrease. An emerging inference was that based on the insights accruing from the assessment of the cannabis group, there was better sleep and appetite, and adverse effects were not observed or reported. Despite the potential effectiveness of cannabinoids in such an investigation, whether the cannabis group would still experience both anti-inflammatory effects and clinical improvements concurrently remained unknown. Rather, most observations centered on the clinical improvement aspect.

Dried cannabis flowers in cigarettes have also been analyzed to determine their effect in 32 patients, including placebo cigarettes and 80 mg tetrahydrocannabinol [16]. With the Lichtiger Index utilized, the results demonstrated clinical remission and also abdominal pain and bowel movement management. Also, patients experienced better quality of life, leading to the inference that cannabinoids come with a great potential for anti-inflammatory effect induction in the pathology. The effects of placebo or oral cannabidiol in patients diagnosed with Crohn’s disease have also been investigated [17]. With 19 participants on the focus, the aim has been to determine the efficacy of the intervention involving 10 mg administered twice each day, centering on individuals with years 39 ± 15 as the mean age. With the period of treatment being eight weeks, findings have been observed to suggest a decrease in CDAI values, with 220 ± 122 recorded on the part of the study group, a decrease from a score of 337 ± 108. Also, within the study group, complete remission was observed in four participants. The safety of cannabis use was also ascertained in the investigation, but situations in which more prominent beneficial effects were not documented. As such, insight into the use of isolated compounds and also the dose used was not vivid.

Discussion

IBD-related predisposing factors include gut microbes, epithelial barrier defects, uncontrolled inflammation, and host genetics. Also, IBD may be caused by exogenous factors in forms such as the Western diet constituting processed meat, prepackaged food items, animal products, and high-fat dairy products. In IBD, therefore, the factors play a crucial role. Some of the key symptoms of the condition include weight loss as an exaggerated response relative to microbial antigens, rectal bleeding, diarrhea, and abdominal pain. In patients with a familial disposition, the implication is that such changes, unless addressed, may lead to intestinal mucosa damage.

Certain treatment modalities have been established to help in IBD management, including immunosuppressants and anti-inflammatories [11]. However, the majority of these treatment options come with certain side effects. Some of these side effects include infusion or injection reactions, malignancies, and opportunistic infections. As such, alternative and complementary medicine has become more and more popular, examples being high-fiber diets, probiotics, vitamins, and prebiotics [12]. One of the additional potential therapeutic options that have been explored is the case of Cannabis sativa and its derivatives.

From the systematic review documented in Table 1 above, the studies’ significant findings and points of convergence demonstrated general well-being improvement in IBD patients, as well as the quality of life improvement, deemed statistically significant. Also, the health perception of patients improved in most patients, as well as better outcomes relative to CDAI levels, complete remission, improvement in weight, and disease activity. It can be inferred, however, that patients are likely to be more tolerant of a placebo than a CBD-rich derivative. When it comes to the factor of well-being, the results point to the potential of cannabinoids to bring about a decrease in the number of bowel movements. Cannabis has been shown to increase gastrointestinal motility and promotion of bowel movements.

Following treatment with cannabis cigarettes, the results depict further that there tends to be a better patient perception of their general health, proving statistically significant and reflecting the promising effects of using cannabinoids in addressing IBD. Simultaneous improvements reported suggest that the use of cannabinoids yields notable improvements in IBD patients’ social functioning, as well as improvements in the parameters of depression, body pain, and the ability to work. It can be seen also that the administration of cannabis via cigarettes that contain tetrahydrocannabinol comes with the promising effect of yielding complete remission as depicted by positive outcomes in changes in the CDAI scores. In cannabis groups, therefore, the specific positive outcomes are seen to evolve in terms of better sleep and appetite, weaning from dependency on steroids, and minimal cases of significant side effects. With changes in blood count less likely, therefore, it is evident that the use of cannabinoids is associated with higher satisfaction from the treatment, improved appetite, and decreased physical pain.

However, when managing CD of moderate severity, no benefits have been documented in some cases, despite the safety of cannabinol. The safety has been highlighted to entail fewer or no cases of changes in or side effects such as liver and kidney function tests, albumin, or hemoglobin. What is worth noting is that direct evidence concerning cannabis’s best protective dose, when used with IBD patients, is unclear. Whereas patients relying on cigarettes with 50 mg plants that are dry-processed have not had side effects impairing their working ability, CD remission induction has not been realized in selected cases, especially after using dried cannabis flowers. These mixed outcomes confirm the inconclusive debate concerning the extent to which cannabinoids may be beneficial for IBD patients, as well as the dilemma concerning the best protective dose worth recommending.

From the observations above, this study established that cannabis, as per the initial results accruing from selected studies, yields promising outcomes, but its social and legal implications in relation to unmonitored use imply that the product needs to be allowed only with caution even at a time when its benefits for individuals with IBD have been documented. Whereas more and more reports suggest that cannabinoids come with a promising effect regarding clinical implications and also the ability to ameliorate gastrointestinal symptoms, the scientific evidence proving the extent to which cannabis modifies or may modify IBD disease objectively remains insufficient. The implication for future scientific research is that there is a need to focus on the recording of IBD patients’ endoscopic healing and biomarker profiles, hence definitive outcome documentation. Similarly, it is recommended that in the future, more and more in vivo and in vitro studies are conducted, as well as an increase in the number of extensive trials accompanied by proper randomization to foster a better understanding of the extent to which cannabinoids could play a protective role in IBD patients.

It is also notable that on the one hand, promising results of cannabinoids have been established in the form of reductions in clinical complications, Mayo scores, CDAI scores, weight gain, health perception enhancement, Lichtiger Index, and general well-being or Harvey-Bradshaw Index. On the other hand, the medical use of cannabinoids in IBD patients remains questionable especially because of the lack of studies deemed to be reporting evidence of high quality. The eventuality is that during future scientific research, it will be key to engage in the designing of safer cannabinoid derivatives that could be used in IBD patients, particularly through randomized clinical trials involving larger sample sizes to arrive at more informed and valid, and reliable outcomes. By implementing these recommendations, the appropriate dose might be determined, as well as the side effects, protective effects, and mode of administration of cannabis, eventually informing the decision for or against accepting the product as an ideal IBD therapeutic product.

Limitations

One limitation is that the findings were characterized by a state of high heterogeneity, implying that outcome generalizability from the review was highly likely to be restricted. Factors that depicted the heterogeneity of the findings included the study designs of the studies that were selected or included, disease activity indices, the duration of treatment by different scholarly researchers, the difference in the modes of administration of cannabinoids and cannabis by different researchers, variations in cannabis dosage, differences in the selected studies’ inclusion criteria, and variations in their case definitions. Lastly, the number of RCTs was limited, with the inclusion of observational research implying that the risk of detection and selection biases was likely to be higher, which could also reduce the strength of the outcomes.

## Conclusions

This systematic review established that although the efficacy of cannabinoid use in IBD treatment was reported in most studies, outcome generalizability from the review was highly likely to be restricted. In addition, very few adverse effects were documented but these documentations were not enough to form opinions on the adverse effect profile for cannabinoid and cannabis use in patients with IBD, including UC and CD. Also, follow-up data post-study was inadequate. However, the selected studies’ point of convergence is that they confirmed the promising role of cannabinoids in steering improvements in IBD treatment through some objective clinical rating scales such as weight gain, Harvey-Bradshaw Index, Mayo score, CDAI score, and general well-being. They also report very limited clinical complications following treatment with cannabis. In the future, the focus of research should be on treatment parameters associated with cannabis and its derivatives to validate its effectiveness and safety in IBD treatments as well as confirm the ideal doses, dose intervals, and routes of administration in IBD treatment based on patient characteristics such as age, gender, and the severity of IBD symptoms.

## References

[1] Pellino G, Keller DS, Sampietro GM (2020). Inflammatory bowel disease position statement of the Italian Society of Colorectal Surgery (SICCR): ulcerative colitis. Tech Coloproctol.

[2] Mak JY, So J, Tang W (2020). Cancer risk and chemoprevention in Chinese inflammatory bowel disease patients: a population-based cohort study. Scand J Gastroenterol.

[3] Li X, Li M, Zheng R, Chen X, Xiang J, Wu FX, Wang J (2019). Evaluation of pathway activation for a single sample toward inflammatory bowel disease classification. Front Genet.

[4] Hansen TM, Sabourin BC, Oketola B, Bernstein CN, Singh H, Targownik LE (2020). Cannabis use in persons with inflammatory bowel disease and vulnerability to substance misuse. Inflamm Bowel Dis.

[5] Papamichael K, Cheifetz AS, Irving PM (2020). New role for azathioprine in case of switching anti-TNFs in IBD. Gut.

[6] Kafil TS, Nguyen TM, MacDonald JK, Chande N (2020). Cannabis for the treatment of Crohn’s disease and ulcerative colitis: evidence from Cochrane Reviews. Inflamm Bowel Dis.

[7] Downer EJ (2020). Anti-inflammatory potential of terpenes present in Cannabis sativa L. ACS Chem Neurosci.

[8] Weinberger AH, Zhu J, Lee J, Anastasiou E, Copeland J, Goodwin RD (2020). Cannabis use among youth in the United States, 2004-2016: faster rate of increase among youth with depression. Drug Alcohol Depend.

[9] Andersen AM, Lei MK, Beach SR, Philibert RA, Sinha S, Colgan JD (2020). Cigarette and cannabis smoking effects on GPR15+ Helper T cell levels in peripheral blood: relationships with epigenetic biomarkers. Genes (Basel).

[10] Kienzl M, Storr M, Schicho R (2020). Cannabinoids and opioids in the treatment of inflammatory bowel diseases. Clin Transl Gastroenterol.

[11] Becker W, Alrafas HR, Wilson K (2020). Activation of cannabinoid receptor 2 prevents colitis-associated colon cancer through myeloid cell de-activation upstream of IL-22 production. iScience.

[12] Wang C, Cui B, Sun Y, Wang C, Guo M (2022). Preparation, stability, antioxidative property and in vitro release of cannabidiol (CBD) in zein-whey protein composite nanoparticles. LWT.

[13] Borrelli F, Fasolino I, Romano B (2013). Beneficial effect of the non-psychotropic plant cannabinoid cannabigerol on experimental inflammatory bowel disease. Biochem Pharmacol.

[14] Irving PM, Iqbal T, Nwokolo C (2018). A randomized, double-blind, placebo-controlled, parallel-group, pilot study of cannabidiol-rich botanical extract in the symptomatic treatment of ulcerative colitis. Inflamm Bowel Dis.

[15] Naftali T, Bar-Lev Schleider L, Dotan I, Lansky EP, Sklerovsky Benjaminov F, Konikoff FM (2013). Cannabis induces a clinical response in patients with Crohn's disease: a prospective placebo-controlled study. Clin Gastroenterol Hepatol.

[16] Naftali T, Bar-Lev Schleider L, Scklerovsky Benjaminov F, Konikoff FM, Matalon ST, Ringel Y (2021). Cannabis is associated with clinical but not endoscopic remission in ulcerative colitis: a randomized controlled trial. PLoS One.

[17] Naftali T, Mechulam R, Marii A (2017). Low-dose cannabidiol is safe but not effective in the treatment for Crohn's disease, a randomized controlled trial. Dig Dis Sci.

[18] Nallathambi R, Mazuz M, Ion A (2017). Anti-inflammatory activity in colon models is derived from Δ9-tetrahydrocannabinolic acid that interacts with additional compounds in cannabis extracts. Cannabis Cannabinoid Res.

[19] Cocetta V, Governa P, Borgonetti V (2021). Cannabidiol isolated from Cannabis sativa L. protects intestinal barrier from in vitro inflammation and oxidative stress. Front Pharmacol.

[20] Couch DG, Cook H, Ortori C, Barrett D, Lund JN, O'Sullivan SE (2019). Palmitoylethanolamide and cannabidiol prevent inflammation-induced hyperpermeability of the human gut in vitro and in vivo-a randomized, placebo-controlled, double-blind controlled trial. Inflamm Bowel Dis.

[21] Pagano E, Iannotti FA, Piscitelli F (2021). Efficacy of combined therapy with fish oil and phytocannabinoids in murine intestinal inflammation. Phytother Res.

[22] Silvestri C, Pagano E, Lacroix S (2020). Fish oil, cannabidiol and the gut microbiota: an investigation in a murine model of colitis. Front Pharmacol.

[23] Singh UP, Singh NP, Singh B, Price RL, Nagarkatti M, Nagarkatti PS (2012). Cannabinoid receptor-2 (CB2) agonist ameliorates colitis in IL-10(-/-) mice by attenuating the activation of T cells and promoting their apoptosis. Toxicol Appl Pharmacol.

[24] Pagano E, Romano B, Iannotti FA (2019). The non-euphoric phytocannabinoid cannabidivarin counteracts intestinal inflammation in mice and cytokine expression in biopsies from UC pediatric patients. Pharmacol Res.

[25] Hoffenberg EJ, McWilliams SK, Mikulich-Gilbertson SK (2018). Marijuana use by adolescents and young adults with inflammatory bowel disease. J Pediatr.

[26] Matarazzo AP, Elisei LS, Carvalho FC, Bonfílio R, Ruela AM, Galdino G, Pereira GR (2021). Mucoadhesive nanostructured lipid carriers as a cannabidiol nasal delivery system for the treatment of neuropathic pain. Eur J Pharm Sci.

[27] Harvey BS, Sia TC, Wattchow DA, Smid SD (2014). Interleukin 17A evoked mucosal damage is attenuated by cannabidiol and anandamide in a human colonic explant model. Cytokine.

[28] Tartakover Matalon S, Azar S, Meiri D (2021). Endocannabinoid levels in ulcerative colitis patients correlate with clinical parameters and are affected by cannabis consumption. Front Endocrinol (Lausanne).

[29] Nso N, Nyabera A, Nassar M (2021). Cannabis and its potential protective role against inflammatory bowel disease: a scoping review. Cureus.

